# Growth Differentiation Factor 15 Regulates Oxidative Stress-Dependent Ferroptosis Post Spinal Cord Injury by Stabilizing the p62-Keap1-Nrf2 Signaling Pathway

**DOI:** 10.3389/fnagi.2022.905115

**Published:** 2022-07-04

**Authors:** Mingjie Xia, Qinyang Zhang, Yanan Zhang, Rulin Li, Tianyu Zhao, Lingxia Chen, Qiangxian Liu, Shengnai Zheng, Haijun Li, Zhanyang Qian, Lei Yang

**Affiliations:** ^1^Department of Orthopedics, Nanjing First Hospital, Nanjing Medical University, Nanjing, China; ^2^Postgraduate School, Dalian Medical University, Dalian, China; ^3^Department of Orthopedics, Taizhou People’s Hospital, Nanjing Medical University, Taizhou, China; ^4^Department of Cardiology, Nanjing University of Chinese Medicine, Nanjing, China; ^5^Taizhou Clinical Medical School of Nanjing Medical University, Taizhou, China; ^6^Department of Orthopedics, Zhongda Hospital of Southeast University, Nanjing, China; ^7^School of Medicine, Southeast University, Nanjing, China; ^8^School of Biomedical Engineering and Informatics, Nanjing Medical University, Nanjing, China

**Keywords:** spinal cord injury, GDF15, oxidative stress, ferroptosis, p62-Keap1-Nrf2 pathway

## Abstract

**Background:**

Spinal cord injury (SCI) is a severe traumatic disorder of the central nervous system (CNS) that causes irreversible damage to the nervous tissue. The consequent hemorrhage contributed by trauma induces neuronal ferroptosis post SCI, which is an important death mode to mediate neuronal loss. Growth differentiation factor 15 (GDF15) is a cytokine that regulates cell proliferation, differentiation, and death. However, the specific role of GDF15 in neuronal ferroptosis post SCI remains unknown.

**Materials and Methods:**

Neuronal ferroptosis *in vitro* was measured by detection of lipid peroxidation, glutathione, iron content, and reactive oxidative stress. *In vivo*, western blotting and immunofluorescence (IF) staining was utilized to measure ferroptosis post SCI. IF staining, TUNEL staining, hematoxylin-eosin staining, and Nissl staining were used to measure neurological damage. Finally, locomotor function recovery was analyzed using the Basso Mouse Scale and Louisville Swim Scale.

**Results:**

GDF15 was significantly increased in neuronal ferroptosis and silencing GDF15 aggravated ferroptosis both *in vitro* and *in vivo*. Besides, GDF15-mediated inhibition of neuronal ferroptosis is through p62-dependent Keap1-Nrf2 pathway. In SCI mice, knockdown of GDF15 significantly exacerbated neuronal death, interfered with axon regeneration and remyelination, aggravated ferroptosis-mediated neuroinflammation, and restrained locomotor recovery.

**Conclusion:**

GDF15 effectively alleviated neuronal ferroptosis post SCI *via* the p62-Keap1-Nrf2 signaling pathway and promoted locomotor recovery of SCI mice, which is suggested as a potential target on SCI pathogenesis and treatment.

## Introduction

Spinal cord injury (SCI) is an extremely serious traumatic disease leading to high rates of mortality and disability (Ahuja et al., 2017; Rong et al., 2021b). Following trauma, the disruption of microvessels causes erythrocyte leakage, which produces excess iron (Silva et al., 2014; Yu et al., 2022). The latter insults neurons, contributing to the intracellular iron metabolism disorder and the generation of reactive oxygen species (ROS), which destroy cellular functions and lead to ferroptosis (Zhang et al., 2022). Consequently, a large amount of neuronal loss caused by ferroptosis affects motor and sensory function post SCI (Huang et al., 2020; Feng et al., 2021). However, the intact mechanism of neuronal ferroptosis after SCI remains elusive.

Ferroptosis is an iron-dependent type of programmed cell death that was put forward by Dixon in 2012 (Dixon et al., 2012; Yang and Stockwell, 2016), of which the nature is oxidative damage, accumulation of iron-stimulated ROS, and lipid peroxidation (Mou et al., 2019). Ferroptosis is involved in the pathogenesis of multiple central nervous system (CNS) diseases like Alzheimer’s disease and subarachnoid hemorrhage (Lane et al., 2021; Qu et al., 2021). Interestingly, ferroptosis also plays an important role in mouse models of SCI, and inhibition of ferroptosis attenuates damage to nervous tissue and promotes neuronal functional recovery (Li et al., 2017; Zille et al., 2017; Chen et al., 2020).

Growth and differentiation factor 15 (GDF15), a cytokine of the transforming growth factor-β (TGF-β) superfamily, is associated with various pathophysiological processes (Emmerson et al., 2018; Wang et al., 2021). GDF15 plays a multifunctional role in proliferation, apoptosis, aging, inflammatory response, and malignancy (Luan et al., 2019; Tarfiei et al., 2019; Borner et al., 2020). In addition, evidence demonstrated that GDF15 was related to the level of hepcidin, which could degrade iron transporter ferroportin (Jiang et al., 2014). Although GDF15 has been investigated in various diseases, whether it has an impact on regulating the pathological process of oxidative stress-dependent neuronal ferroptosis after SCI remains elusive.

In this study, we found that GDF15 was significantly increased in neuronal ferroptosis both *in vitro* and *in vivo* and that silencing GDF15 aggravated neuronal ferroptosis. Furthermore, we first demonstrated that GDF15 inhibits oxidative stress-dependent ferroptosis in neurons post SCI through the p62-Keap1-Nrf2 signaling pathway and alleviates neurological damage, which consequently promotes locomotor function recovery in SCI mice. Therefore, GDF15 is suggested as a potential target on regulating neuronal ferroptosis, and our findings may provide a new insight into SCI pathogenesis and treatment.

## Materials and Methods

### Extraction and Culture of Primary Neurons

The primary neurons were extracted from the cerebral cortex of fetal mice according to a previous study (Pu et al., 2020). Briefly, an 18-day pregnant mouse was anesthetized and a cesarean was performed, then its fetal mice were sacrificed in 75% ethanol. The cerebral cortex was removed from the fetal mice, digested with 2 mg/ml papainase (Sigma-Aldrich, St. Louis, MO, United States) dissolved in Dulbecco’s modified Eagle medium (DMEM; KeyGEN, Nanjing, China) for 30 min, then the mixed cell group suspended in DMEM supplemented 10% fetal bovine serum (FBS; Gibco, Grand Island, NY, United States) were seeded in 6-well plates (Corning, NY, United States). After 4 h culture, the neurons were cultured with the neurobasal medium (Gibco) supplemented 1% glutamine and B27 (Gibco). Then the primary neurons were treated with Hemin (100 μM, MedChemExpresss, Weehawken, NJ, United States) for 24 h.

### Spinal Cord Injury Model

Total of 50 C57BL/6J adult mice (males, average weight of 20 g, 8 weeks of age) were acquired from Charles River (Beijing, China). All mice were given free food and water and housed at 50% humidity as well as 22°C ± 1°C temperature with a 12 h/12 h light-dark cycle. The operation of SCI was the same as our previous study (Jiang et al., 2021). Our animal protocol was approved by the Institutional Animal Care and Use Committee of Southeast University (Approval No.: 20210302042). In brief, ketamine (80 mg/kg) was utilized to anesthetize mice before the skin was prepared for disinfection, and then the back skin was incised to expose the lamina at T10. Finally, a moderate contusion (5 g × 5 cm) was created by an impactor (RWD, Shenzhen, China). Spinal cord hemorrhage, hindlimb extension, and delayed paralysis suggest successful modeling. Only laminectomy was performed in the Sham group.

### Analysis of Cell Viability

Cell viability was assessed by Cell Counting Kit-8 (CCK-8) assay (Biosharp, Hefei, China). Primary neurons were treated with different concentrations of Hemin for 24 h and CCK-8 (10 μl) was added in neurons for a 1 h incubation at 37°C. The absorbance was detected by a microplate reader (BioTek Instruments, Inc., Winooski, VT, United States) at 450 nm.

### Regulation of Growth Differentiation Factor 15 and p62 Expression

The short hairpin (sh) RNAs targeting GDF15 and p62 (BIOG, Changzhou, China) for knockdown were loaded in plasmids, respectively, and transfected into primary neurons by RFect Plasmid Transfection Reagent (BIOG) for 24 h. Recombinant GDF15 (rGDF5; ab202199, Abcam, Cambridge, MA, United States) was used to provide exogenetic expression of GDF15 *in vitro* (Li H. et al., 2021). The shRNA-GDF15 loaded in adeno-associated virus (AAV) vectors was obtained from Genechem CO., LTD (Shanghai, China). The AAV containing shRNA-GDF15 were delivered into mice at 1 week before SCI modeling *via* the intracortical injections (Lewandowski and Steward, 2014). Briefly, the scalp was clipped and a small piece of skull was removed after anesthesia. Then, 1 μl of AAVshRNA-GDF15 (10^8^ genome copies/ml) was injected 0.8 mm underneath the brain surface at 1 mm anterior to bregma and 2 mm lateral to the midline.

### Real-Time Quantitative Reverse-Transcription PCR

A TRIzol reagent (YiFeiXue Biotechnology, Nanjing, China) was employed to extract RNA of spinal cords according to the manufacturer’s guidance. After the RNA concentration was measured, a reverse transcription kit and a qPCR Kit (YiFeiXue Biotechnology) were utilized to carry out qRT-PCR in the Roche LightCycler 480 (Roche, Basel, Switzerland). The primer sequences of GDF15, p62, and GAPDH were as follows: GDF15: forward: CTGGCAATGCCTGAACAACG; reverse: GGTCGGGACTTGGTTCTGAG; p62: forward: GAGGCAC CCCGAAACATGG; reverse: ACTTATAGCGAGTTCCCACCA; GAPDH: forward: TGACCTCAACTACATGGTCTACA; reverse: CTTCCCATTCTCGGCCTTG.

### Western Blotting

Total protein from the primary neurons and the spinal cords was extracted using a Total Protein Extraction Kit (Keygen). The concentrations of proteins were determined by Enhanced BCA Protein Assay Kit (Beyotime, Shanghai, China). Total of 60 μg proteins in each group were used for WB analysis. The main primary and secondary antibodies used in WB are as follows: anti-GDF15 (1:1,000, 32005, Singalway Antibody, CollegePark, MD, United States), anti-ACSL4 (1:1,000, ab155282, Abcam), anti-FTH-1 (1:1,000, 32180, Singalway Antibody), anti-GPX4 (1:1,000, ab125066, Abcam), anti-p62 (1:10,000, ab109012, Abcam), anti-Keap1 (1:1,000, 41626, Singalway Antibody), anti-Nrf2 (1:1,000, 66504, Proteintech, Rosemount, IL, United States), anti-HO-1 (1:1,000, 86806, Cell Signaling Technology, Boston, MA, United States), anti-GAPDH (1:10,000, HRP-60004, Proteintech), anti-β-Tubulin (1:10,000, HRP-66240, Proteintech), and HRP Goat-anti-Rabbit secondary antibody (1:10,000, YFSA02, YiFeiXue Biotechnology). The bands were captured using a Gel Document System (SYNGENE, Cambridge, United Kingdom), and the protein density was then analyzed using the ImageJ software (National Institutes of Health, Bethesda, MD, United States).

### Lipid Peroxidation, Glutathione, and Iron Content Detection

MDA and 4-HNE, the main products of lipid peroxidation, were measured by their detection kits (S0131M, Beyotime, and ab238538, Abcam) (Rong et al., 2021a). Briefly, for MDA detection, the samples were mixed with the working solution pre-prepared, then heated at 100°C for 15 min and cooled to room temperature. The absorbance at 532 nm was measured under a microplate reader (BioTek). For 4-HNE detection, after adding 50 μl of the diluted anti-4-HNE antibody and 100 μl diluted secondary antibody to samples, respectively, and incubating for 1 h, we washed the mixture and stopped the reaction. The absorbance at 450 nm was detected on a microplate reader (BioTek). The relative GSH level was tested by the GSH assay kit (S0053, Beyotime). Analogously, the sample was mixed with the GSH working solution, and the absorbance at 405 nm was measured on a microplate reader (BioTek). The relative Fe^2+^ concentration was detected by an iron assay kit (ab83366, Abcam). For iron detection, 5 μl of assay buffer was added to each sample and then incubated at 37°C for 30 min, followed by adding 100 μl iron probe to the mixture and incubated at 37°C for 60 min in the dark. Finally, the absorbance at 593 nm was measured on a microplate reader (BioTek).

### Detection of Reactive Oxygen Species

DCFH-DA (YFX0707, YiFeiXue Biotechnology) was diluted with serum-free medium to reach the final concentration of 10 μmol/L. The cell culture medium was replaced by the DCFH-DA solution, followed by a 20 min incubation at 37°C. Washed three times by serum-free medium, the neurons were collected and resuspended by PBS for flow cytometry (FACSVerse 8, BD).

### Immunofluorescence Staining

Neurons and paraffin sections of cords were incubated with primary antibodies overnight at 4°C after blocking with immunol staining blocking buffer (Beyotime) for 1 h, followed by incubation with fluorescent secondary antibodies in the dark for 1 h. The antibodies used were as follows: anti-GDF15 (1:100, 32005, Singalway Antibody), anti-ACSL4 (1:100, ab155282, Abcam), anti-p62 (1:1,000, ab109012, Abcam), anti-Nrf2 (1:100, 66504, Proteintech), anti-GPX4 (1:100, ab125066, Abcam), anti-NeuN (1:100, ab177487; Abcam), anti-IBA-1 (1:500, ab178847; Abcam), anti-GFAP (1:600, 3670; Cell Signaling Technology), anti-NF200 (1:200, ab82259; Abcam), anti-MBP (1:600, ab7349; Abcam), 488 AffiniPure Fab Fragment Goat-anti-Rabbit secondary antibody (1:500, 111-547-003, Jackson ImmunoResearch, PA, United States), and 594 AffiniPure Fab Fragment Goat Anti-Rabbit secondary antibody (1:500, 111-587-003, Jackson ImmunoResearch). After counterstaining with diaminobenzidine (DAPI), the samples were observed under a fluorescent microscope (Leica, Oskar, Germany).

### TUNEL Staining

The death of neurons in injured spinal cords was detected by a TUNEL Staining Kit (Servicebio, Wuhan, China). Briefly, after treating with 0.1% TritonX-100 (Biosharp) for 20 min, the paraffin slices of spinal cords were incubated with the mixture including terminal deoxynucleotidyl transferase (TDT, Servicebio) enzyme, deoxyuridine triphosphates (dUTP, Servicebio), and buffer at a ratio of 1:5:50 at 37°C for 2 h. The sections were counterstained with DAPI and observed under a fluorescence microscope (Leica).

### Hematoxylin-Eosin Staining

The integrity of nervous tissue was assessed with an H&E Staining Kit (Servicebio). Briefly, after paraffin sections were dewaxed, H&E was used to stain nuclei and cytoplasm.

### Nissl Staining

A Nissl Staining Reagent (Servicebio) was utilized to measure the number of neurons in spinal cords at 7 or 28 days post injury (dpi). Briefly, Toluidine Blue was used to stain the sections for 2–5 min, and 1% glacial acetic acid was added. Then, the sections were washed, mounted with neutral balm, and observed under a microscope (Leica).

### Behavioral Assessment

The Basso Mouse Scale (BMS) and Louisville Swim Scale (LSS) were used to assess the locomotor function of mice hindlimbs post SCI (Basso et al., 2006; Smith et al., 2006). BMS scores ranged from 0 (no ankle movement) to 9 (normal movement). LSS had 15 gradings to assess forelimb dependency and hindlimb function (Kong et al., 2020). All the mice in the study were assessed in an open field or a water tank at 1, 3, 7, 14, 21, and 28 dpi.

### Statistical Analysis

The experimental data were exhibited as the mean ± standard deviation (SD) values and analyzed using the Prism software, version 8.3 (GraphPad, San Diego, CA, United States). Comparisons between two groups were analyzed by unpaired *t*-tests, and among more than two groups using one-way or two-way ANOVAs followed by Tukey’s *post hoc* test. *P*-value < 0.05 were regarded as significance.

## Results

### Growth Differentiation Factor 15 Was Increased in Spinal Cord Injury and Neuronal Ferroptosis

**FIGURE 1:**
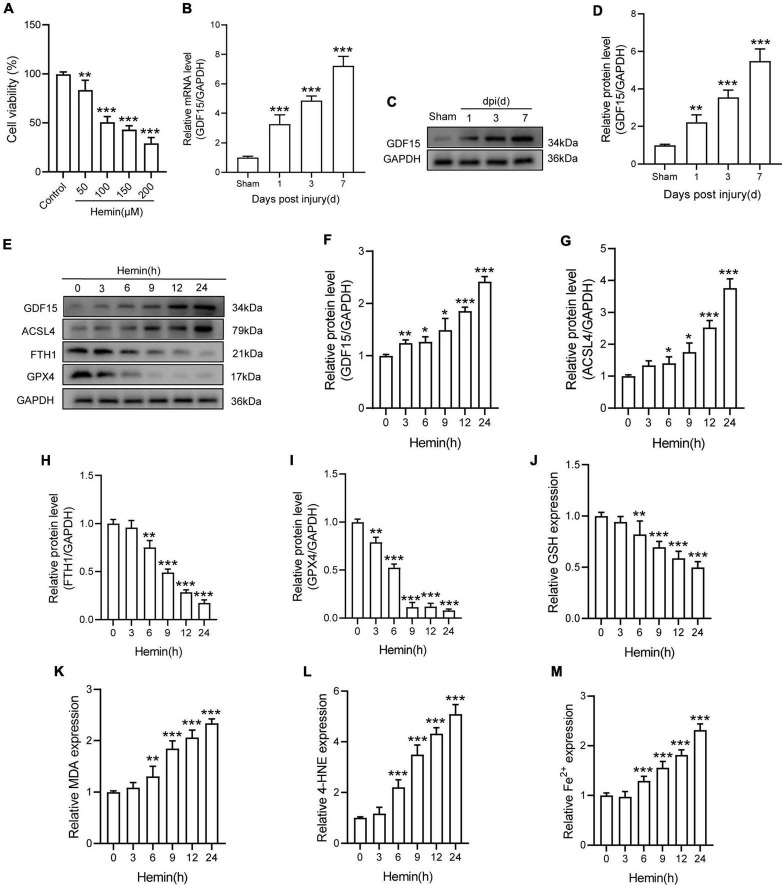
GDF15 was increased in SCI and neuronal ferroptosis. **(A)** Cell viability was detected by CCK-8 (*n* = 6). **(B)** Relative mRNA level of GDF15 in the spinal cord within a week post-injury (*n* = 6). **(C)** Western blotting of GDF15 protein levels in the spinal cord within a week post-injury (*n* = 6). **(D)** Bar graph showing a quantitative analysis of GDF15 expression (*n* = 6). **(E)** Western blotting of GDF15 and ferroptosis-associated proteins including ACSL4, FTH1, and GPX4 in Hemin-stimulated primary neurons within 24 h (*n* = 3). GAPDH was used as the control. **(F–I)** Bar graph showing quantitative analysis of GDF15, ACSL4, FTH1, and GPX4 (*n* = 3). **(J–M)** The values of GSH, MDA, 4-HNE, and Fe^2+^ concentrations were determined; *n* = 6. The error bars represent the SD. *p < 0.05 vs. control group by one-way ANOVA followed by Tukey’s *post hoc* analysis (*p < 0.05, **p < 0.01, and ***p < 0.001). Sham: Only laminectomy was performed.

Ferroptosis inducer Hemin was used to treat with primary neurons for 24 h. The result showed that Hemin obviously decreased the cell viability (Figure 1A). Besides, qRT-PCR and WB analysis revealed that both the mRNA and protein expressions of GDF15 were increasing within a week post SCI and peaked at 7 dpi (Figures 1B–D). To further determine the expression of GDF15 in neuronal ferroptosis, we treated the neurons with Hemin within 24 h *in vitro*, finding that GDF15 significantly upgraded after Hemin treatment (Figures 1E,F). In addition, the ferroptosis-related protein ACSL4 was increased but the other two markers FTH1 and GPX4 decreased after Hemin treatment (Figures 1G–I). Then, we measured the lipid peroxidation, GSH, and iron content in Hemin-treated neurons medium. The results exhibited that the expression of GSH markedly reduced, which linked to the obviously increased Fe^2+^ content as well as the lipid peroxidation products MDA and 4-HNE after Hemin treatment, indicating that neuronal ferroptosis was effectively stimulated (Figures 1J–M).

### Growth Differentiation Factor 15 Effectively Alleviated Oxidative Stress-Dependent Neuronal Ferroptosis *in vitro*

**FIGURE 2:**
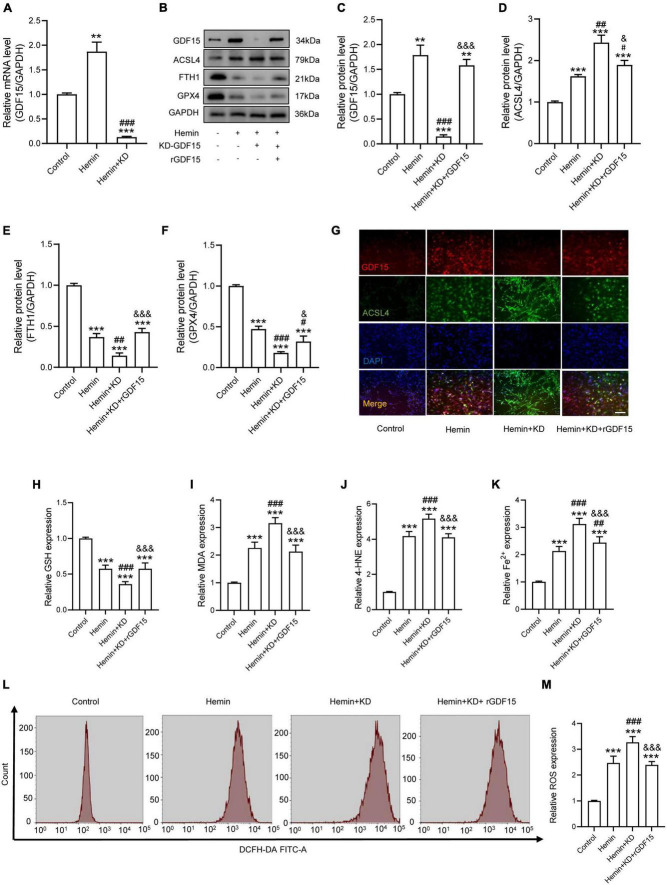
GDF15 effectively alleviated oxidative stress-dependent neuronal ferroptosis *in vitro*. **(A)** Relative mRNA level of GDF15 after knockdown (*n* = 6). **(B)** Western blotting performed for GDF15 and ferroptosis-associated proteins including ACSL4, FTH1, and GPX4 in Hemin-activated primary neurons after transfection of KD-GDF15 or adding rGDF15 (*n* = 3). GAPDH was used as the control. **(C–F)** Bar graph showing quantitative analysis of GDF15, ACSL4, FTH1, and GPX4 (*n* = 3). **(G)** Representative immunofluorescence labeling images for GDF15 (red) and ACSL4 (green) in Hemin-activated primary neurons after transfection of KD-GDF15 or adding rGDF15 (Scale bar = 50 μm). **(H–K)** The values of GSH, MDA, 4-HNE, and Fe^2+^ concentrations were determined (*n* = 6). **(L)** The value of ROS was determined (*n* = 6). **(M)** Bar graph showing quantitative analysis of ROS expression (*n* = 6). The error bars represent the SD. **p < 0.01, ***p < 0.001, vs. control group; ^#^*p* < 0.05, ^##^*p* < 0.01, ^###^*p* < 0.001, vs. Hemin group; ^&^*p* < 0.05, ^&⁣&&^*p* < 0.001, vs. Hemin + KD group by one-way ANOVA followed by Tukey’s *post hoc* analysis.

To research the function of GDF15 in oxidative stress-induced neuronal ferroptosis, we transfected neurons with plasmids loaded with sh-GDF15 to knock down GDF15 expression (KD-GDF15). The mRNA and protein levels of GDF15 were significantly decreased after knockdown compared to control group (Figures 2A–C). WB results revealed that the expression of ACSL4 increased more, but FTH1 and GPX4 decreased more in KD-GDF15 neurons compared to the untreated neurons after Hemin insult. However, the above results were relieved by rGDF15 supplement (Figures 2D–F). IF staining also showed that rGDF15 increased the expression of ACSL4 inhibited by KD-GDF15 (Figure 2G). Additionally, GSH level was significantly reduced, but the expression of MDA, 4-HNE, and Fe^2+^ were increased markedly after KD-GDF15 treatment compared with Hemin group, whereas the results were reversed by rGDF15 (Figures 2H–K). The detection of ROS also confirmed the above results (Figures 2L,M).

### Growth Differentiation Factor 15 Mitigates Hemin-Induced Reactive Oxygen Species Production and Ferroptosis in Neurons Through the p62-Keap1-Nrf2 Signaling Pathway

**FIGURE 3:**
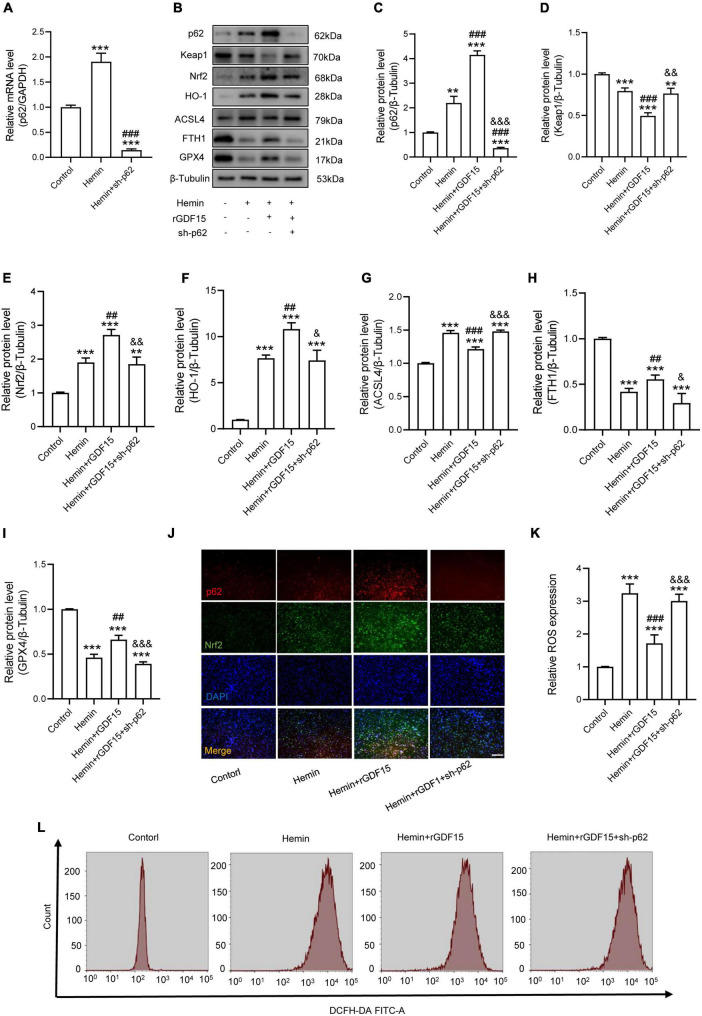
GDF15 mitigates Hemin-induced ROS production and ferroptosis in neurons through the p62-Keap1-Nrf2 signaling pathway. **(A)** Relative mRNA level of p62 after knockdown (*n* = 6). **(B)** Western blotting performed for p62, Keap1, Nrf2, HO-1, ACSL4, FTH1, and GPX4 in Hemin-activated primary neurons after adding rGDF15 or transfection of sh-p62 (*n* = 3). β-Tubulin was used as the control. **(C–I)** Bar graph showing quantitative analysis of p62, Keap1, Nrf2, HO-1, ACSL4, FTH1, and GPX4 (*n* = 3). **(J)** Representative immunofluorescence labeling images for p62 (red) and Nrf2 (green) in Hemin-activated primary neurons after adding rGDF15 or transfection of sh-p62 (Scale bar = 200 μm). **(K)** Bar graph showing quantitative analysis of ROS expression (*n* = 6). **(L)** The value of ROS was determined (*n* = 6). The error bars represent the SD. **p < 0.01, ***p < 0.001, vs. control group; ^##^*p* < 0.01, ^###^*p* < 0.001, vs. Hemin group; ^&^*p* < 0.05, ^&&^*p* < 0.01, ^&⁣&&^*p* < 0.001, vs. Hemin + rGDF15 group by one-way ANOVA followed by Tukey’s *post hoc* analysis.

The P62-Keap1-Nrf2 signaling axis has been reported to be associated with ferroptosis (Ren et al., 2021). We next investigated whether GDF15 inhibited ferroptosis by regulating the p62-Keap1-Nrf2 signaling pathway. We transfected sh-p62 to hemin-treated neurons to silence p62 expression, and both the mRNA and protein levels of p62 were markedly decreased compared with control group (Figures 3A,B). In agreement with previous studies, our results showed that the p62-Keap1-Nrf2 signaling axis was activated with ferroptosis occurring after Hemin treatment. In addition, rGDF15 further increased the expression of p62, followed by the decrease of Keap1 and increase of Nrf2 and HO-1, which indicated that GDF15 promoted the activation of the p62-Keap1-Nrf2 signaling pathway and alleviated oxidative stress in neuronal ferroptosis (Figures 3C–F). Besides, in consistent with previous findings, rGDF15 reduced the level of ferroptosis; however, ferroptosis level was increased again when p62 was knocked down, which showed that GDF15-mediated inhibition of ferroptosis was p62-dependent (Figures 3G–I). We next used IF to detect the expression of p62 and Nrf2 in neuronal ferroptosis. The results displayed that rGDF15 increased the protein level of p62 and Nrf2 compared to Hemin-treated group, which was in accordance with WB results (Figure 3J). In addition, the ROS level was prominently decreased by treatment with rGDF15, whereas that reversely increased after the knockdown of p62 (Figures 3K,L).

### Silencing Growth Differentiation Factor 15 Aggravated Ferroptosis After Spinal Cord Injury *via* Destabilizing p62 and Nrf2 Level

**FIGURE 4:**
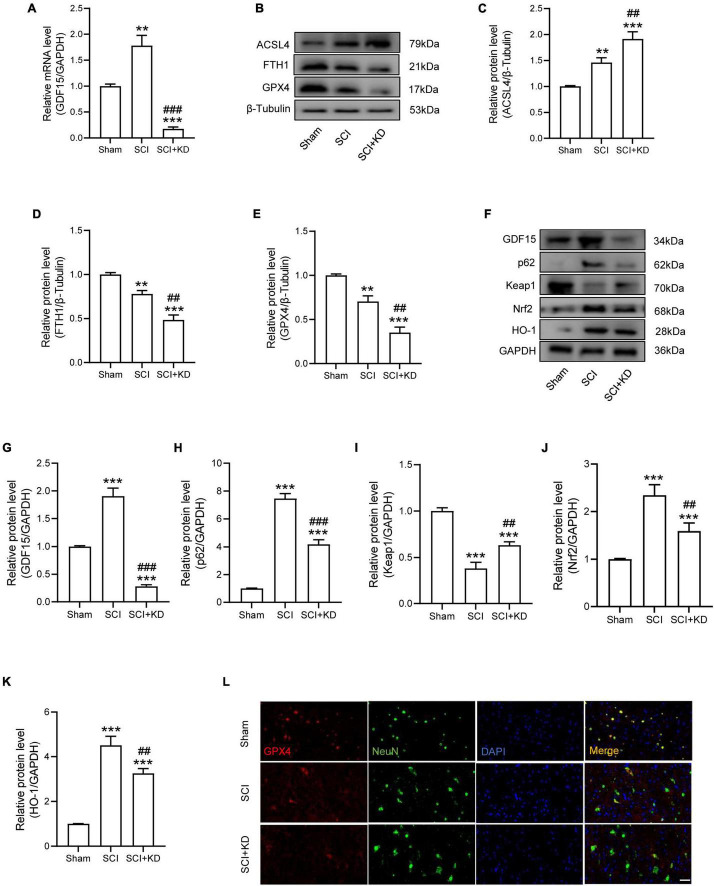
Silencing GDF15 aggravated ferroptosis after SCI *via* destabilizing p62 and Nrf2 level. **(A)** Relative mRNA level of GDF15 in SCI mice after knockdown (*n* = 6). **(B)** Western blotting of ACSL4, FTH1, and GPX4 protein levels at 1 dpi in Sham, SCI, and SCI + KD mice (*n* = 3). **(C–E)** Bar graph showing a quantitative analysis of ACSL4, FTH1, and GPX4 (*n* = 3). **(F)** Western blotting performed for GDF15, p62, Keap1, Nrf2, and HO-1 at 1 dpi in Sham, SCI, and SCI + KD mice (*n* = 3). **(G–K)** Bar graph showing a quantitative analysis of GDF15, p62, Keap1, Nrf2, and HO-1 (*n* = 3). **(L)** Double IF of GPX4-1 (red) and NeuN (green), obtained from longitudinal sections centered around central canal at 1 dpi in Sham, SCI, and SCI + KD mice (Scale bar = 40 μm). The error bars represent the SD. **p < 0.01, ***p < 0.001, vs. Sham group; ^##^*p* < 0.01, ^###^*p* < 0.001, vs. SCI group by one-way ANOVA followed by Tukey’s *post hoc* analysis.

We next researched the role of GDF15 in SCI mice. The AAV containing shRNA-GDF15 was injected to mice, and the mRNA expression of GDF15 was significantly decreased compared to Sham group (Figure 4A). At 1 dpi, WB revealed that SCI obviously increased the protein expression of ACSL4 and decreased the protein expression of GPX4 and FTH1 when compared with the Sham group. Notably, KD-GDF15 further deteriorated ferroptosis (Figures 4B–E). Furthermore, the p62-Keap1-Nrf2 signaling pathway was activated after SCI. However, KD-GDF15 significantly decreased the protein level of p62, Nrf2, and HO-1 but increased the protein level of Keap1, which indicated that SCI-induced ferroptosis was deteriorative (Figures 4F–K). We next used IF staining of GPX4 and NeuN to detect neuronal ferroptosis after SCI. The results displayed that the expression of GPX4 in neurons was reduced after SCI, and silencing GDF15 caused the further reduction of GPX4 expression in neurons (Figure 4L).

### Inhibition of Growth Differentiation Factor 15 Aggravated Neurological Damage and Neuroinflammation

**FIGURE 5:**
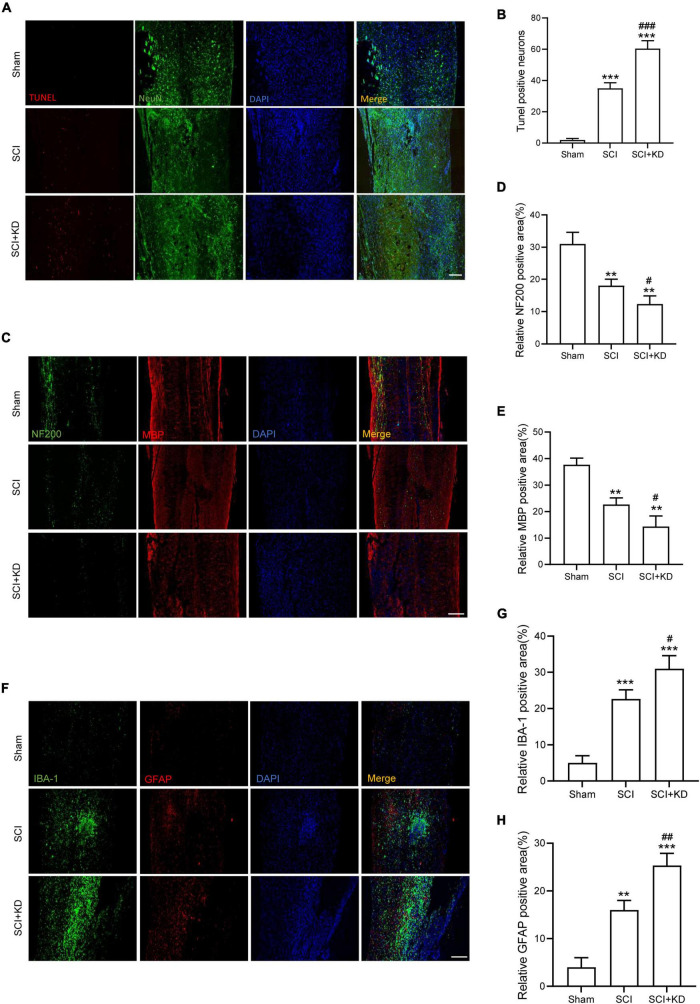
Inhibition of GDF15 aggravated neurological damage and neuroinflammation. **(A)** Neuronal death determined by TUNEL assay at 7 dpi in Sham, SCI, and SCI + KD mice (Scale bar = 200 μm). **(B)** Quantitative analysis of TUNEL-positive neurons. **(C)** Representative immunofluorescence labeling of neurofilaments for NF200 (green) and myelin sheath for MBP (red) and obtained from longitudinal sections centered around the injured core 1.5 mm at 28 dpi (Scale bar = 300 μm). **(D)** Quantitative analysis of NF200 positive area at 28 dpi (*n* = 6). **(E)** Quantitative analysis of MBP positive area at 28 dpi (*n* = 6). **(F)** Double immunofluorescence labeling of microglia for IBA-1 (green) and astrocytes for GFAP (red) obtained from longitudinal sections centered around the injured core 3 mm at 7 dpi (Scale bar = 300 μm). **(G)** Quantitative analysis of IBA-1 positive area at 7 dpi. **(H)** Quantitative analysis of GFAP positive area at 7 dpi. The error bars represent the SD. **p < 0.01, ***p < 0.001, vs. Sham group; ^#^*p* < 0.05, ^##^*p* < 0.01, ^###^*p* < 0.001, vs. SCI group by t-test, one-way ANOVA followed by Tukey’s *post hoc* analysis.

At 7 dpi, TUNEL staining results showed that Tunel-positive neurons was significantly increased after SCI and KD-GDF15 further aggravated neuronal death (Figures 5A,B). Additionally, KD-GDF15 caused lower levels of axon numbers and myelin sheath numbers labeled by NF-200 and MBP, respectively, when compared with SCI group, which indicated axon regeneration and remyelination, were significantly inhibited (Figures 5C–E). Previous studies have reported that excessive accumulation of iron promoted the activation of microglia and thus exacerbated neuroinflammation (Wang et al., 2022). We have verified silencing GDF15 aggravated ferroptosis after SCI, and we next analyzed whether GDF15 affected ferroptosis-mediated neuroinflammation. Neuroinflammation after SCI is characterized by high expression of activated microglia and astrocytes (Qian et al., 2022); our results showed that both the expressions of IBA-1, a biological marker of microglia, glial fibrillary acidic protein (GFAP), and a characteristic marker of astrocytes were significantly increased when inhibition of GDF15 compared with SCI only, which suggested that silencing GDF15 further aggravated the ferroptosis-mediated neuroinflammation (Figures 5F–H).

### Knockdown of Growth Differentiation Factor 15 Interferes Locomotor Recovery by Aggravated Neuronal Loss in Spinal Cord Injury Mice

**FIGURE 6:**
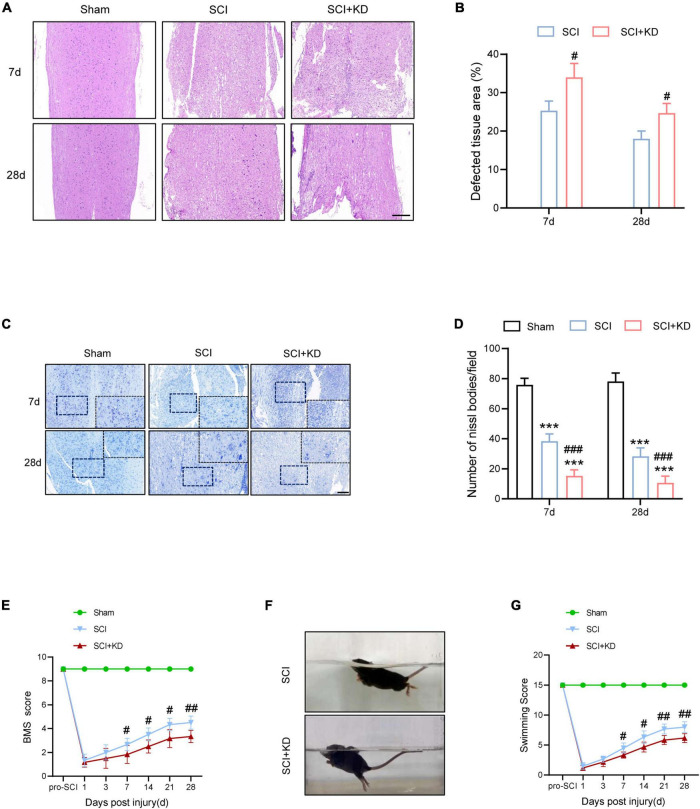
Knockdown of GDF15 interferes locomotor recovery by aggravated neuronal loss in SCI mice. **(A)** H&E staining images of cords centered around the injured core 3 mm obtained at 7 and 28 dpi (Scale bar = 300 μm). **(B)** Quantitative analysis of the defected area at 7 and 28 dpi (*n* = 6). **(C)** Representative images for Nissl staining obtained from longitudinal sections centered around the injured core 1.5 mm at 7 and 28 dpi in Sham, SCI, and SCI + KD mice (Scale bar = 200 μm). **(D)** Quantitative analysis of the amounts of survived neurons at 7 and 28 dpi (*n* = 6). **(E)** The BMS score post SCI in Sham, SCI, and SCI + KD mice. **(F,G)** Photographs of Swimming at 28 dpi, showing the worse trunk instability and uncoordinated action in SCI mice, and statistical analysis of the Louisville Swim Scale over a period of 28 days (*n* = 6). ***p < 0.001, vs. Sham group; ^#^*p* < 0.05, ^##^*p* < 0.01, ^###^*p* < 0.001, vs. SCI group by two-way ANOVA followed by Tukey’s *post hoc* analysis.

At 7 and 28 dpi, H&E staining showed more defective tissue area in KD-GDF15 mice compared to SCI alone, indicating that knockdown of GDF15 aggravated nervous tissue loss post SCI (Figures 6A,B). Additionally, Nissl staining also demonstrated that SCI caused neuronal loss compared with the Sham group, which was further deteriorated by KD-GDF15 (Figures 6C,D). The BMS and LSS were employed to evaluate the locomotor recovery of SCI mice. The results showed low scores within 3 days post SCI. Interestingly, the scores of KD-GDF15 mice were significantly less than the SCI mice starting on 7 dpi and lasting until 28 dpi (Figure 6E). The LSS also displayed that the KD-GDF15 SCI mice exhibited worse body balance, weaker hindlimb alternation, and larger body-surface angle starting at 7 dpi. Briefly, silencing of GDF15 led to more neuronal loss and consequently affected locomotor recovery post SCI (Figures 6F,G).

## Discussion

In this study, we first reported the functional role of GDF15 in inhibiting oxidative stress-dependent neuronal ferroptosis post SCI by regulating ferroptosis levels through targeting the p62-Keap1-Nrf2 signaling pathway. Neuronal death and the damage of nervous tissue have always been the severe pathologic process of SCI that significantly impairs motor and sensory function of patients and causes high mortality and bring great burden to the patients and their families (Li C. et al., 2021; Haque et al., 2022; Valeri et al., 2022). Ferroptosis has been previously reported a form of neuronal death in SCI. The pathogenesis of cell rupture, hemorrhaging, hemolysis, and supernumerary iron post SCI caused excess ROS accumulation, which results in ferroptosis occurrence (Hu et al., 2021; Ge et al., 2022). Our results showed that ferroptosis-related protein GPX4, primarily expressed in neurons, significantly decreased while ACSL4 increased both *in vitro* and *in vivo*. Hence, inhibiting ferroptosis is a central link to alleviate neuronal death after SCI.

Previous studies have reported that GDF15, a cytokine associated with cell growth and differentiation, might effectively predicated the prognosis of colorectal cancer patients as a ferroptosis-related gene (Shao et al., 2021). Besides, GDF15 plays a vital role in metabolic disorders (Chow et al., 2022), inflammatory process (Sanchez-Infantes et al., 2021), tumor progression (Jin et al., 2021), and interestingly, neurodegenerative diseases (Rochette et al., 2020). Therefore, GDF15 may mediate the processes of iron metabolism and thus affect the occurrence and development of ferroptosis in CNS disorders. However, the mechanism of GDF15 in neuronal ferroptosis after SCI remains unknown. To examine the effect of GDF15 in SCI, we detected the level of GDF15 in injured spinal cords and the results displayed that both mRNA and protein levels of GDF15 were significantly elevated post SCI. Interestingly, we found that GDF15 protein levels were also significantly increased in neuronal ferroptosis *in vitro*. We then showed that knockdown of GDF15 significantly aggravated ferroptosis, which was rescued by rGDF15. This also identified that GDF15 is closely linked to ferroptosis. We demonstrated that GDF15 alleviates neuronal ferroptosis after SCI, but its specific regulatory mechanism needs to be further studied.

As what mentioned before, SCI is a complex pathologic process involving neuronal ferroptosis with iron-induced lipid peroxidation and production of large amounts of ROS (Ebrahimi et al., 2022; Liao et al., 2022). Exploration of potential intervention targets to ferroptosis is promising for the treatment of many clinical diseases. The p62-Keap1-Nrf2 signaling axis was reported to effectively inhibit ROS accumulation and oxidative stress thus alleviating ferroptosis (Hou et al., 2021; Zhao et al., 2021; Yuan et al., 2022). Sun et al. (2016) have reported that activating the p62-Keap1-Nrf2 axis can effectively inhibit ferroptosis in liver cancer. Mechanically, Keap1 inhibits Nrf2 expression through ubiquitination under normal conditions. When ferroptosis occurs, increasing p62 promotes the autophagy degradation of Keap1, which facilitates Nrf2 to be released into the nucleus. Nrf2 activates downstream transcription factors such as HO-1, which further restrains the production of ROS, thus inhibiting ferroptosis (Sun et al., 2020; Zhang et al., 2021). In agreement with previous studies, we found that the p62-Keap1-Nrf2 signaling axis was stimulated in neuronal ferroptosis both *in vitro* and *in vivo*. Markedly, rGDF15 treatment further promotes the expression of p62, followed by the increased protein levels of Nrf2 and HO-1 *in vitro*. Oppositely, knockdown of GDF15 in SCI mice significantly inhibited the p62-Keap1-Nrf2 signaling pathway and consequently aggravated ferroptosis. To examine the regulatory mechanism between GDF15 and p62, we silenced p62 on the basis of rGDF15 and found that not only the activation of the pathway was inhibited, but also the level of ferroptosis was conversely increased. These results also suggested that GDF15 reduced ferroptosis by regulating the expression of p62.

**FIGURE 7:**
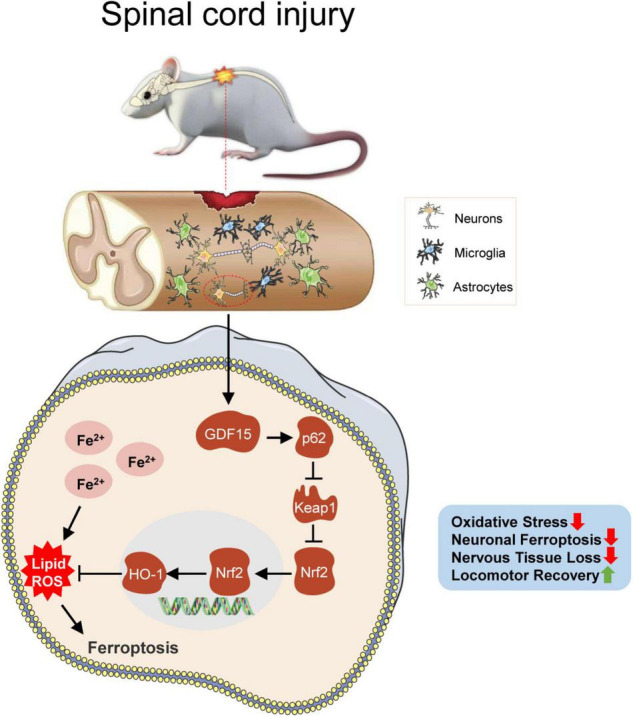
GDF15 alleviates SCI-induced neuronal ferroptosis by regulating the p62-Keap1-Nrf2 signaling pathway. After SCI, cracked blood vessels at the injured cords result in the lysis and destruction of erythrocytes, which release a large amount of iron. Excessive iron accumulated in neurons produces superfluous ROS and causes neuronal ferroptosis, which promotes the activation of the p62-Keap1-Nrf2 signaling pathway. Besides, GDF15 in neurons further facilitates the activation of the p62-Keap1-Nrf2 pathway by stabilizing p62 and increased Nrf2 and HO-1 inhibit the accumulation of ROS and thus alleviate neuronal ferroptosis. GDF15 is suggested as a potential target on mitigating nervous tissue loss and promoting locomotor recovery post SCI.

In this study, neuronal ferroptosis was observed after SCI. We sought to investigate the neuroprotective role of GDF15 post SCI and found that the death of neurons was more severe after knockdown of GDF15 at both 7 and 28 dpi. Deteriorative loss of neurons can lead to inevitable damage to nervous tissue followed by demyelination and motor system disorders (Feng et al., 2021; Rong et al., 2021a). We found that silencing GDF15 impeded axonal regeneration and remyelination and aggravated nervous tissue loss post SCI. Previous studies have shown that overloaded iron-induced ferroptosis promoted the activation of microglia by a ROS-independent mechanism and the latter caused the secretion of pro-inflammatory cytokines such as tumor necrosis factor-α (TNF-α) and interleukin-1β (IL-1β), which triggered neuroinflammation (Ko et al., 2021). In our study, we observed that after deteriorative ferroptosis caused by the knockdown of GDF15, neuroinflammation post SCI was also more severe, which suggested that GDF15 was associated with ferroptosis-mediated neuroinflammation. However, the specific role of GDF15 in neuroinflammation post SCI needs to be further researched. Finally, both BMS and LSS showed delayed locomotor recovery in KD-GDF15 mice, which demonstrated that KD-GDF15 further inhibited the recovery of motor function in SCI mice by aggravating neuronal ferroptosis (Figure 7).

## Conclusion

Growth differentiation factor 15 is a neuroprotective factor that regulates oxidative stress-dependent ferroptosis post SCI by stabilizing the p62-Keap1-Nrf2 signaling pathway. Silencing GDF15 aggravates neuronal ferroptosis, increases nervous tissue damage, and interferes with locomotor recovery in SCI mice. Our results revealed the specific role of GDF15 in neuronal ferroptosis, which may be a promising treatment target for SCI. However, other regulatory effects of GDF15 involved in neuronal ferroptosis and neuroinflammation after SCI remain uncertain. Thus, further studies of GDF15 in SCI need to be implemented in the future.

## Data Availability Statement

The original contributions presented in this study are included in the article/supplementary material, further inquiries can be directed to the corresponding authors.

## Ethics Statement

The animal study was reviewed and approved by the Institutional Animal Care and Use Committee of Southeast University (Approval No. 20210302042).

## Author Contributions

LY, ZQ, and MX conceived and designed the experiments. MX, QZ, YZ, RL, and TZ performed the experiments. SZ analyzed the data. MX wrote the manuscript. QL and LC maintained the mice colonies. HL and LY funded and supervised the study. All authors read and approved the final manuscript for publication.

## Conflict of Interest

The authors declare that the research was conducted in the absence of any commercial or financial relationships that could be construed as a potential conflict of interest.

## Publisher’s Note

All claims expressed in this article are solely those of the authors and do not necessarily represent those of their affiliated organizations, or those of the publisher, the editors and the reviewers. Any product that may be evaluated in this article, or claim that may be made by its manufacturer, is not guaranteed or endorsed by the publisher.
